# MicroRNA expression profile of chicken cecum in different stages during *Histomonas meleagridis* infection

**DOI:** 10.1186/s12917-022-03316-2

**Published:** 2022-06-11

**Authors:** Yu-Ming Zhang, Qiao-Guang Chen, Chen Chen, Shuang Wang, Zai-Fan Li, Zhao-Feng Hou, Dan-Dan Liu, Jian-Ping Tao, Jin-jun Xu

**Affiliations:** 1 College of Veterinary Medicine, Yangzhou University, Yangzhou, 225009 People’s Republic of China; 2 Jiangsu Co-innovation Center for Prevention and Control of Important Animal Infectious Diseases and Zoonosis, Yangzhou, 225009 People’s Republic of China

**Keywords:** Host regulation, microRNA, Chicken, Cecum, *Histomonas meleagridis*, Inflammation, Immune

## Abstract

**Background:**

*Histomonas meleagridis* is an anaerobic, intercellular parasite, which infects gallinaceous birds such as turkeys and chickens. In recent years, the reemergence of Histomoniasis has caused serious economic losses as drugs to treat the disease have been banned. At present, *H. meleagridis* research focuses on virulence, gene expression analysis, and the innate immunity of the host. However, there are no studies on the differentially expressed miRNAs (DEMs) associated with the host inflammatory and immune responses induced by *H. meleagridis*. In this research, high-throughput sequencing was used to analyze the expression profile of cecum miRNA at 10 and 15 days post-infection (DPI) in chickens infected with Chinese JSYZ-F strain *H. meleagridis.*

**Results:**

Compared with the controls, 94 and 127 DEMs were found in cecum of infected chickens at 10 DPI (CE vs CC) and 15 DPI (CEH vs CCH), respectively, of which 60 DEMs were shared at two-time points. Gene Ontology (GO) functional enrichment analysis of the target genes of DEMs indicated that 881 and 1027 GO terms were significantly enriched at 10 and 15 DPI, respectively. Kyoto Encyclopedia of Genes and Genomes (KEGG, www.kegg.jp/kegg/kegg1.html) pathway enrichment analysis of the target genes of DEMs demonstrated that 5 and 3 KEGG pathways were significantly enriched at 10 and 15 DPI, respectively. For previous uses, the Kanehisa laboratory have happily provided permission. The integrated analysis of miRNA–gene network revealed that the DEMs played important roles in the host inflammatory and immune responses to *H. meleagridis* infection by dynamically regulating expression levels of inflammation and immune-related cytokines.

**Conclusion:**

This article not only suggested that host miRNA expression was dynamically altered by *H. meleagridis* and host but also revealed differences in the regulation of T cell involved in host responses to different times *H. meleagridis* infection.

**Supplementary Information:**

The online version contains supplementary material available at 10.1186/s12917-022-03316-2.

## Background

Histomoniasis, also known as infectious cecum hepatitis or “blackhead disease”, is a disease of gallinaceous birds (turkeys, chickens, quails, and peacocks) caused by the *H. meleagridis* protozoan parasite [1]. *H. meleagridis* has a complex life history and transmission routes [2]. In the natural environment, it usually parasitizes in *Heterakis gallinarum (H. gallinarum*) eggs or earthworm and survive for a long time [3, 4]. *H. meleagridis* mainly parasitize the cecum and liver of the host [1], causing cecum mucosal lesion, intestinal wall hypertrophy, caseous cecum core, and yellowish-green round inflammatory necrotic focus in the liver, which seriously affects the metabolism and absorption of nutrients, in severe cases, excessive inflammatory response and immune dysfunction caused infected avian death [5]. The intestinal mucosal is the host’s first barrier against *H. meleagridis* infection. IgA, an important part of mucosal immunity against pathogen invasion, has been shown that it will be continuously elevated in response to *H. meleagridis’* invasion of cecum mucosa [6]. Presently, Histomoniasis have become a worldwide disease. In Europe and America where farming a large number of turkeys, with high morbidity and fatality [7]. Although chickens are less vulnerable to the disease than turkeys (100%), the epidemic of the disease in Chinese chicken flocks is also very serious, and the mortality rates are 20 to 30% [8]. Since most chemical drugs that can effectively control and prevent the disease were banned owing to their potential carcinogenicity [9], which has contributed to the incidence of the disease is increasing year by year and causing severe economic losses.

MicroRNA (miRNA) is a class of short non-coding RNA molecules expressed by animals, plants, viruses, and some single-celled organisms, with a length of approximately 22 nucleotides [10]. MiRNAs play important role in the regulation of cellular signal networks in both normal and diseased conditions. In eukaryotes, 2/3 of the coding genes are regulated by miRNAs [11], which participate in the regulation of many physiological processes [12], such as development, cell division, proliferation, and metabolism, and play an essential role in the inflammatory response [13], immune-related pathway [14], tumorigenesis [15] and so on. A growing number of studies have shown that host miRNAs modulate target gene expression at the post-transcriptional level by inhibiting translation and promoting degradation of target genes, and play an important role in against parasite infection [16, 17].

Since the first report of Histomoniasis, increased attention has been paid to histopathological features [18], etiology [19], virulence [20], and gene expression analysis [21]. However, the molecular mechanism of the interaction between *H. meleagridis* and chicken is not clear, especially the study of miRNA expression in chicken cecum after *H. meleagridis* infection. This study investigated the expression profile of chicken cecum miRNA at 10 and 15 days post-infection. To our knowledge, this is the first report on the expression of miRNA in chicken cecum during *H. meleagridis* infection. This article will further our understanding of the interactions between the host and *H. meleagridis*. This will aid the development of novel therapies against *H. meleagridis* in the future.

## Methods

### Animals and experimental infection

An F strain of *H. meleagridis,* obtained from a home-bred chicken in Jiangsu Province, China, was cryopreserved in liquid nitrogen in our laboratory. 40 SPF White Leghorn layers (15-day old) were used in this study. 30 chickens were divided into the infection groups and 10 chickens were divided into the control group. Chickens in the infection group were inoculated with 2 × 10^5^ *H. meleagridis* through the cloaca, and the control group chickens were not treated.

### Sample collection and preparation

At 10 and 15 days post-infection (DPI), half of the chickens in the two groups were killed, respectively. The chickens cecum samples aseptically collected, were thoroughly rinsed in PBS, and immediately frozen in liquid nitrogen. All the cecum samples were stored at − 80 °C until RNA extraction [17].

Total RNA was prepared from individual cecum samples using TRIzol Reagent (Invitrogen, Carlsbad, CA, USA) according to the manufacturer’s instruction. The purity and integrity of RNA samples were assessed using the RNA Nano 6000 Assay Kit of the Agilent Bioanalyzer 2100 system (Agilent Technologies, CA, USA) and spectrophotometer (IMPLEN, CA, USA), respectively [17].

### Small RNA library preparation and sequencing

Twelve libraries were constructed from the cecum of 10 DPI (CE), and control group (CC), 15 DPI (CEH), and control group (CCH), with three in each group. The small RNA libraries were prepared from a total of 2 μg total RNA isolated from each sample using NEBNext® Multiplex Small RNA Library Prep Set for Illumina® (NEB, USA) according to the manufacturer’s instruction and sequenced at the Novogene Bioinformatics Institute (Beijing, China) on an Illumina Novaseq 6000 platform (Illumina, San Diego, CA, USA) following the vendor’s instructions.

### Basic data processing

Raw data (raw reads) of Fastq format were firstly processed through custom Perl and Python scripts. In this step, clean data (clean reads) were obtained by removing reads containing ploy-N, ploy A or T or G or C, or with 5′ adapter contaminants, or without 3′ adapter or the insert tag. Moreover, the 3′ adapter sequences were trimmed. At the same time, Q20, Q30, and GC content of the raw data were calculated. Clean reads with 18 to 35 nt length range were chosen for downstream analyses.

The processed small RNA reads were used in Bowtie [22] for read mapping to reference sequence. This allowed for 1 mismatch base.

Processed reads of length at 18 to 35 nt were then mapped to their reference genome and analyzed using the bowtie package (no mismatch). To identify conserved miRNAs, the predicted miRNA hairpins were compared against miRNA precursor sequences from miRBase22.0 (http://www.mirbase.org/) using mirDeep2 [23]. Srna-tools-cli (http://srna-tools.cm.uea.ac.uk/) were used to obtain the potential miRNA and draw the secondary structures. MirDeep2’s quantifier. pl were used to obtain the miRNA counts, and custom scripts were used to obtain base bias on the first position of identified miRNA with 18 to 35 nt length and each position of all identified miRNA respectively.

The available software miREvo [24] and MirDeep2 [23] were integrated to predict novel miRNA through exploring the secondary structure, the Dicer cleavage site and the minimum free energy of the small RNA reads unannotated in the former steps.

All sequence data were submitted to the NCBI Gene Expression Omnibus (GEO) public database (http://www.ncbi.nlm.nih.gov/geo/) with the GEO accession number GSE193859.

### Analysis of differentially expressed miRNAs

The expression levels of known and new miRNAs in each sample were counted, and transcripts per million clean tags (TPM) [25] was used to normalize the expression levels. Differential expression analysis of two groups was performed using the DESeq R package (1.24.0). The *P*-value was adjusted using the Benjamini & Hochberg method [26]. Corrected *P*-value < 0.05 was set as the threshold for screening differentially expressed genes.

### Predicted target genes of miRNAs and bioinformatics analysis

miRanda [27] and RNAhybrid [28] were used to predict the target gene of miRNA. GOseq based Wallenius non-central hypergeometric distribution [29] which could adjust for gene length bias, was implemented for GO enrichment analysis. GO terms with a *P*-value < 0.05 were regarded as significantly enriched terms. Additionally, KEGG [30] pathway with a *P*-value < 0.05 were considered significantly enriched pathways. KOBAS [31] software was used to test the statistical enrichment of the target gene candidates in KEGG pathways.

### miRNA-gene network

To dissect the role of DEMs in inflammatory and immune, Cytoscape3.9.0 software was used to construct DEMs and immune and inflammatory-related genes regulatory networks at 10 and 15 DPI.

### Quantitative real-time qPCR validation

Nine DEMs, including 3 miRNAs in 10 DPI, 2 miRNAs in 15 DPI, and 4 miRNAs shared at 10 and 15 DPI, were selected and measured using SYBR green-based RT-qPCR to verify the sequencing results. miRNA sequences in which uracil was replaced by thymine were used as the forward primers for the real-time PCR described in Table 1. The miRNA primers were synthesized by BGI Co. Ltd. (Shenzhen, China). The total RNA was extracted from cecum samples, and reverse transcribed into cDNA using a Mir-x™ miRNA First-Strand Synthesis and SYBR® RT-qPCR Kit (TaKaRa, Dalian, China) following the manufacturer’s instruction. RT-qPCR cycling conditions were as follows: 95 °C for 5 mins; followed by 45 cycles of 95 °C for 10 s, 60 °C for 10 s, and 72 °C for 15 s; and melting curve analysis from 60 °C to 97 °C. All reactions were carried out with three repeats. U6 snRNA were used as internal reference gene for quantifying miRNA expression analysis. The expression of each miRNA relative to U6 was calculated using the 2 − ΔΔCT method [17].

**Table 1:** The sequences of miRNAs used for RT-qPCR validation

miRNA	Sequence
gga-miR-214	ACAGCAGGCACAGACAGGCAG
gga-miR-34c-5p	AGGCAGUGUAGUUAGCUGAUUGC
gga-miR-17-5p	CAAAGUGCUUACAGUGCAGGUAGU
gga-miR-145-5p	GUCCAGUUUUCCCAGGAAUCCCUU
gga-miR-183	UAUGGCACUGGUAGAAUUCACUG
gga-miR-204	UUCCCUUUGUCAUCCUAUGCCU
gga-miR-2954	CAUCCCCAUUCCACUCCUAGCA
gga-miR-1677-3p	UGACUUCAGUAGGAGCAGGAUU
gga-miR-140-3p	CCACAGGGUAGAACCACGGAC

## Results

### Sequencing of small non-coding RNAs in cecum of *H. meleagridis*-induced chickens

Twelve cecum libraries were constructed from CC, CE, CCH, and CEH groups, with 3 in each group. High-throughput sequencing generated 11,310,686, 11,759,594, 11,103,844, and 12,587,674 average reads in the CE, CC, CEH, and CCH libraries, respectively. After removal of low-quality and adaptor contamination reads, the average clean reads obtained at each group were 11,038,098, 11,527,878, 10,876,563, and 12,315,338 respectively (Table 2).

**Table 2:** The list of data filtering (%)

Library	Sample	total reads	N% > 10%	low quality	5_adapter contamine	3_adapter_null or insert null	with ploy A/T/G/C	clean reads
CE	CE1	11,842,662 (100.00%)	114 (0.00%)	24,351 (0.21%)	556 (0.00%)	299,539 (2.53%)	6660 (0.06%)	11,511,442 (97.20%)
	CE2	10,610,508 (100.00%)	84 (0.00%)	21,567 (0.20%)	628 (0.01%)	230,486 (2.17%)	5824 (0.05%)	10,351,919 (97.56%)
	CE3	11,478,887 (100.00%)	70 (0.00%)	20,039 (0.17%)	538 (0.00%)	201,544 (1.76%)	5762 (0.05%)	11,250,934 (98.01%)
CC	CC1	10,835,812 (100.00%)	98 (0.00%)	12,896 (0.12%)	1523 (0.01%)	75,483 (0.70%)	5689 (0.05%)	10,740,123 (99.12%)
	CC2	12,445,172 (100.00%)	106 (0.00%)	23,116 (0.19%)	1299 (0.01%)	282,358 (2.27%)	8015 (0.06%)	12,130,278 (97.47%)
	CC3	11,997,798 (100.00%)	102 (0.00%)	21,972 (0.18%)	679 (0.01%)	254,882 (2.12%)	6929 (0.06%)	11,713,234 (97.63%)
CEH	CEH1	11,692,196 (100.00%)	0 (0.00%)	62,563 (0.54%)	2126 (0.02%)	259,645 (2.22%)	17,540 (0.15%)	11,350,322 (97.08%)
	CEH2	10,099,875 (100.00%)	0 (0.00%)	43,351 (0.43%)	1199 (0.01%)	118,709 (1.18%)	7777 (0.08%)	9,928,839 (98.31%)
	CEH3	11,519,462 (100.00%)	0 (0.00%)	38,272 (0.33%)	1456 (0.01%)	120,667 (1.05%)	8538 (0.07%)	11,350,529 (98.53%)
CCH	CCH1	13,815,927 (100.00%)	0 (0.00%)	54,488 (0.39%)	2241 (0.02%)	159,117 (1.15%)	37,801 (0.27%)	13,562,280 (98.16%)
	CCH2	13,879,487 (100.00%)	0 (0.00%)	107,019 (0.77%)	3208 (0.02%)	169,015 (1.22%)	81,076 (0.58%)	13,519,169 (97.40%)
	CCH3	10,067,608 (100.00%)	0 (0.00%)	41,304 (0.41%)	1302 (0.01%)	142,941 (1.42%)	17,496 (0.17%)	9,864,565 (97.98%)

**Fig. 1:**
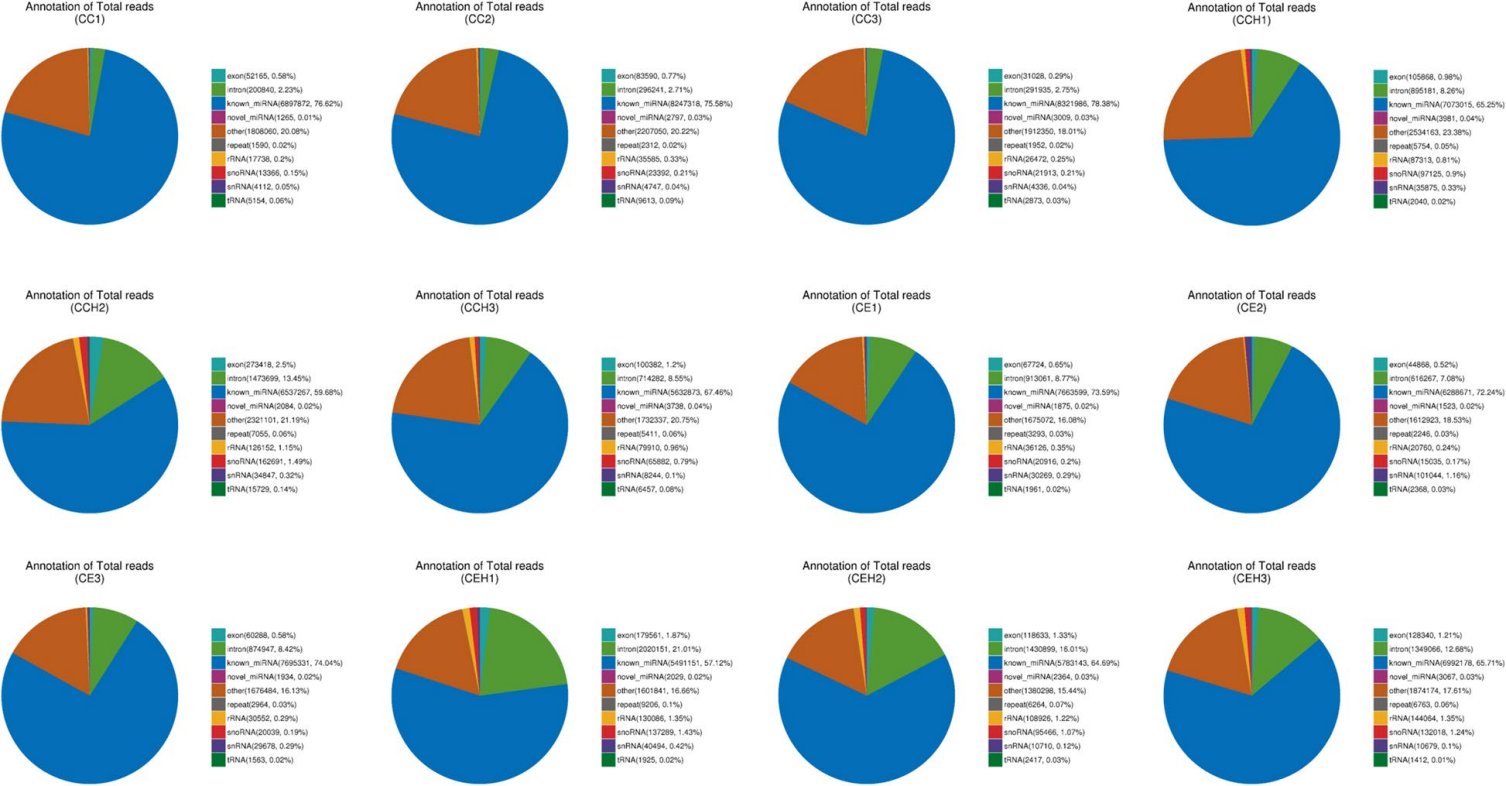
The annotation statistics of the unique reads from samples in each group. ‘other’ represents the unannotated small RNA. (CC1, CC2, CC3 = 10 DPI control groups; CE1, CE2, CE3 = 10 DPI infected groups; CCH1, CCH2, CCH3 = 15 DPI control groups; CEH1, CEH2, CEH3 = 15 DPI infected groups)

Most of the sRNA length in the 12 libraries were 21-24 nt. Among these, the sRNA tags of 83.28–96.43% were mapped to the chicken genome. The repetitive sequence, exon sequence, intron sequence, tRNA, rRNA, snRNA, and snoRNA were successfully annotated. 57.12–78.38% of the reads in each library were identified as known miRNAs, 0.01–0.04% were predicted to be new miRNAs (Fig. 1). A total of 797 known and 91 novel mature miRNAs, corresponding to 667 and 96 precursors, respectively, were identified with a BLAST search against the miRBase or by recognition of standard stem-loop structures (Table 3).

**Table 3:** The known and novel miRNAs mapped in chicken genome

	known miRNAs				novel miRNAs				
Types	Mapped mature	Mapped hairpin	Mapped unique sRNA	Mapped total sRNA	Mapped mature	Mapped star	Mapped hairpin	Mapped unique sRNA	Mapped total sRNA
Total	797	667	63,392	158,535,931	91	28	96	1281	45,044
CE1	400	343	2359	7,663,599	24	2	27	34	1875
CE2	370	320	2152	6,288,671	17	2	19	27	1523
CE3	400	344	2262	7,695,331	17	4	18	29	1934
CC1	354	312	2066	6,897,872	21	1	40	70	2029
CC2	420	362	2529	8,247,318	30	4	33	59	2364
CC3	413	355	2474	8,321,986	31	4	39	66	3067
CEH1	457	410	3013	5,491,151	33	5	30	48	1306
CEH2	452	388	2907	5,783,143	28	6	29	47	1324
CEH3	456	400	2941	6,992,178	35	7	31	49	951
CCH1	456	408	2934	7,073,015	41	4	25	53	2369
CCH2	433	384	2940	6,537,267	32	4	46	80	1803
CCH3	435	385	2797	5,632,873	33	4	58	104	2601

### Differentially expressed miRNAs in cecum of infected and control chickens at different time points

**Fig. 2:**
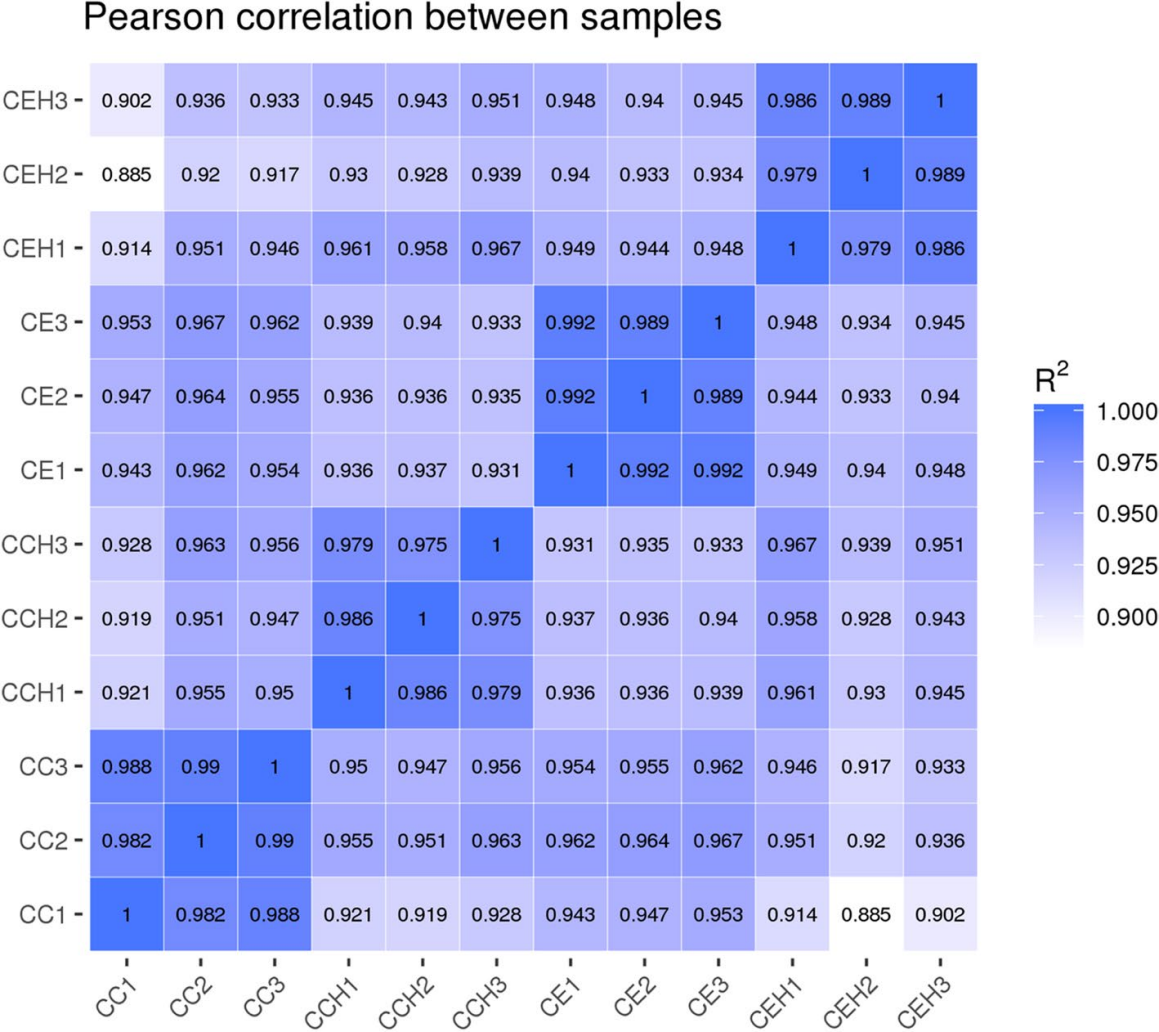
The correlation analysis between samples in different groups. Pearson correlation coefficients were calculated to estimate the association of expression levels between samples

**Fig. 3:**
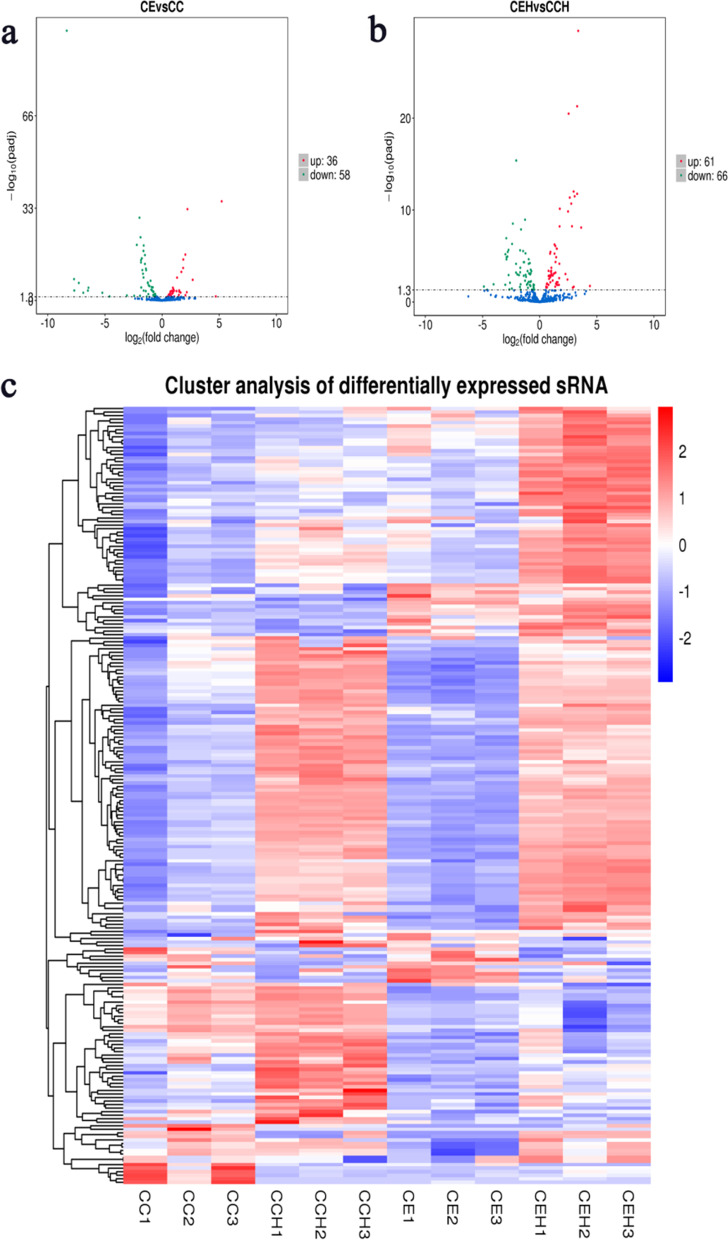
The volcanoplot and heatmap of the differentially expressed miRNAs. **a**, **b** The volcanopiot of the differentially expressed miRNAs at 10 and 15 DPI, respectively. c The heatmap of the differentially expressed miRNAs at 10 and 15 DPI. The blue indicated no significant difference, while the red and green indicated miRNA with significant difference (**a**, **b**). The red indicated higher miRNA expression level and the blue showed lower miRNA expression level (**c**)

**Fig. 4:**
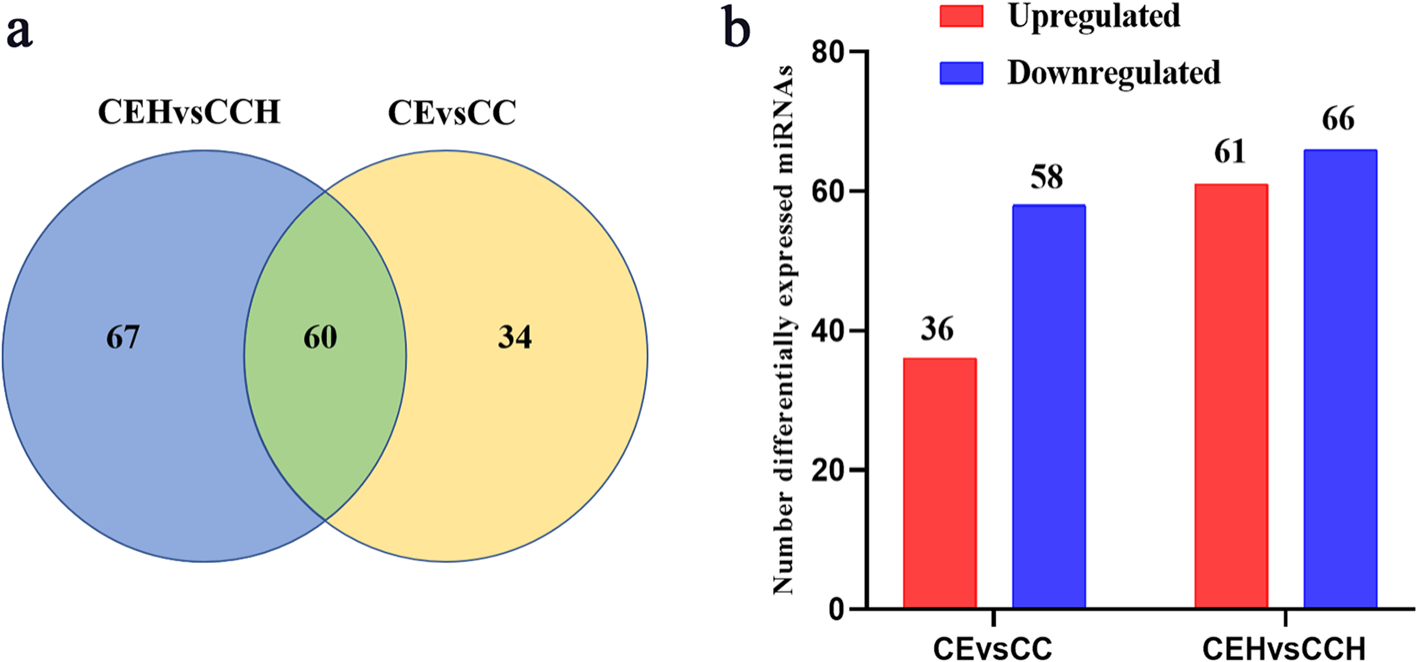
The differential expression analysis of chicken cecal miRNAs between infected and control groups at different periods. **a** The Venn diagrams of the DEMs between the infected and control groups. **b** The number of DEMs between the infected and control groups. (CE vs CC = numbers of 10 DPI DEMs; CEH vs CCH = numbers of 15 DPI DEMs)

Pearson correlation coefficients were used to estimate expression levels to examine the gene expression patterns of miRNAs in different samples. The correlation coefficients ranged from 0.885 for CEH2 versus CC1 to 0.992 for CE1 versus CE2 (Fig. 2). A total of 161 unique chicken-encoded miRNAs were significantly differentially expressed between the infected and control samples at 10 and 15 DPI, including one (gga-novel-123) novel miRNA from 15 DPI. 94 and 127 miRNAs were identified as DEMs at 10 and 15 DPI, respectively (Fig. 3; Additional files 1, 2). A total of 101 DEMs were identified to be sample-specific, including 34 from 10 DPI and 67 from 15 DPI. 60 DEMs were shared at 10 and 15 DPI, of which 24 DEMs were up-regulated and 35 DEMs were down-regulated, interestingly, one (gga-miR-2954) of the DEMs was up-regulated at 10 DPI and down-regulated at 15 DPI (Fig. 4).

### Functional enrichment analysis of target genes of differentially expressed miRNAs

RNAhybrid and Miranda software were used to predict the candidate target genes of each differentially expressed miRNA. A total of 2170 target genes for the 94 DEMs at 10 DPI and 2445 target genes for the 127 DEMs were predicted at 15 DPI. GO functional and KEGG pathway enrichment analyses were performed to better illuminate the functions of the DEMs.

**Fig. 5:**
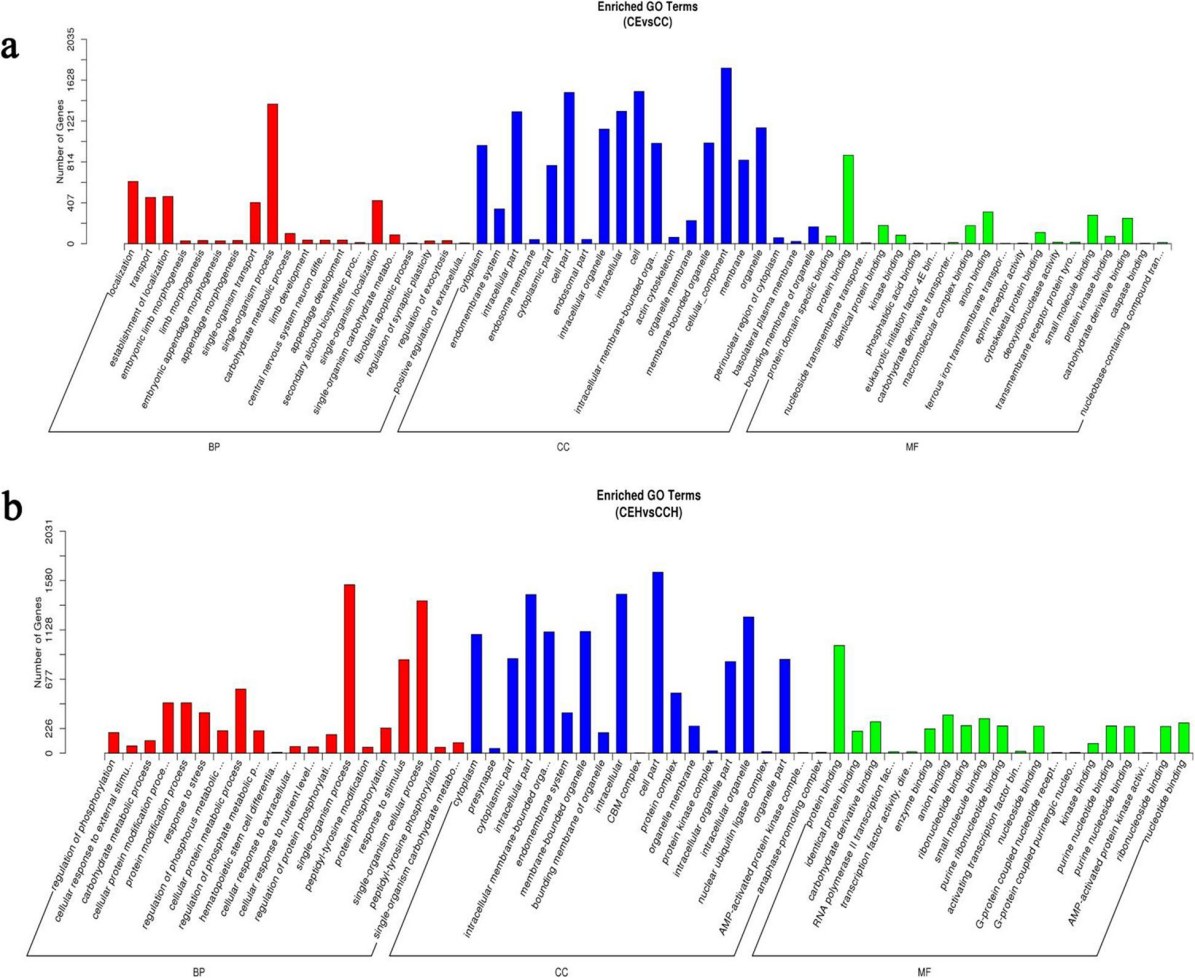
The GO enrichment analysis for target genes of DEMs. **a** The significant enriched GO terms of BP, CC, and MF for target genes of DEMs at CE vs CC. **b** The significant enriched GO terms of BP, CC, and MF for target genes of DEMs at CEH vs CCH. (BP = biological process; CC = cellular component; MF = cellular component)

The GO teams include biological process (BP), cellular component (CC), and molecular function (MF). A total of 881 and 1027 significantly enrichen GO terms (*P* < 0.05) were identified from 10 and 15 DPI, respectively. Some of these GO terms were shared at 10 and 15 DPI, for example, single-organism process, single-organism carbohydrate metabolic process in BP, cytoplasm, intracellular part, an intracellular membrane-bounded organelle in CC, protein binding, anion binding, kinase binding in MF (Fig. 5). Moreover, GO terms related to inflammation (e.g., cell proliferation, inflammatory response, interleukin-6 biosynthetic process, chemokine production) and immune function (e.g., death, response to stimulus, mast cell activation involved in immune response, T cell proliferation, B cell selection) were found at 10 and 15 DPI.

**Fig. 6:**
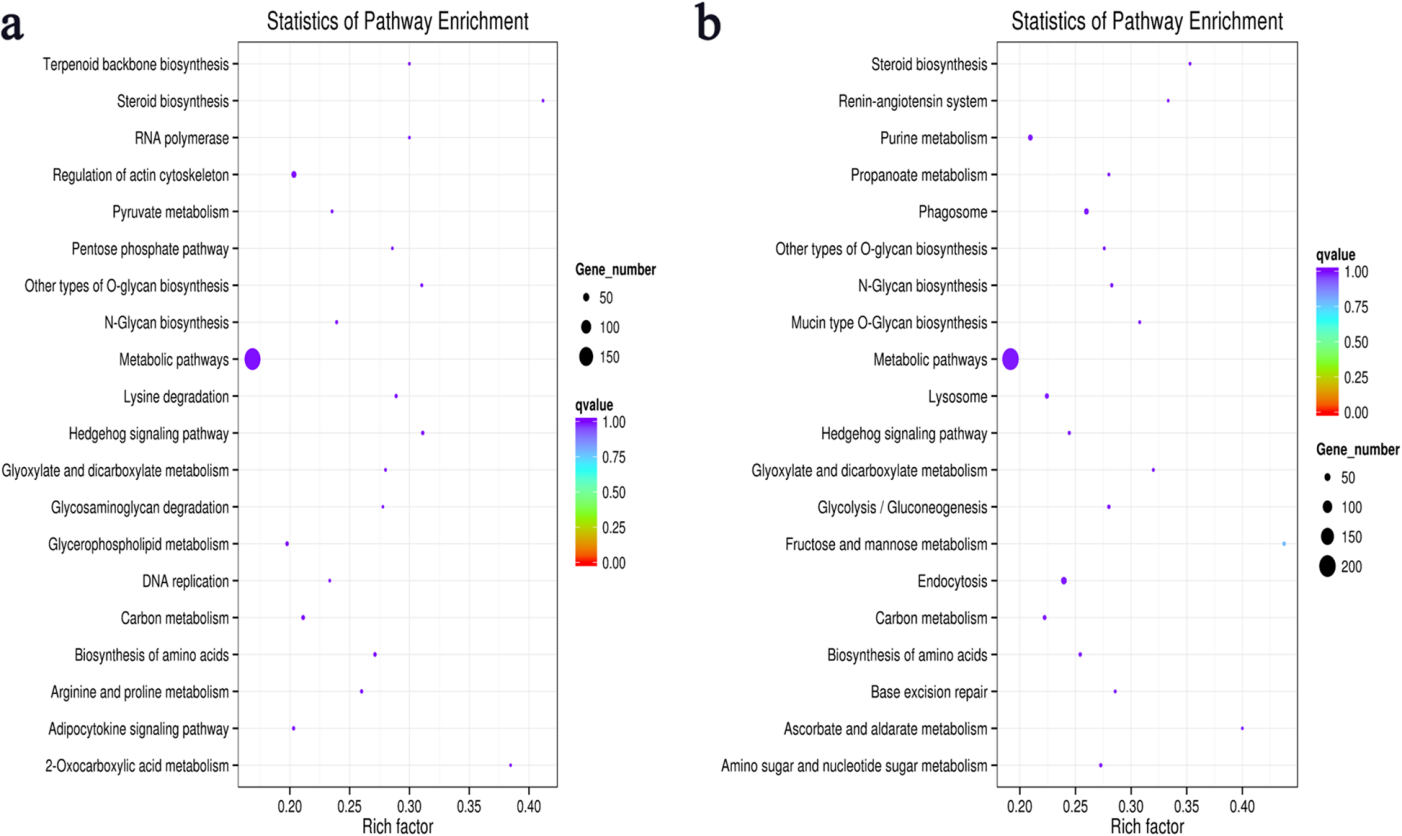
The KEGG pathway analysis for target genes of DEMs. The top 20 KEGG pathways of DEMs at 10DPI **(a)** and 15DPI **(b).** (The point size representss the number of genes enriched in the pathway. The x-axis shows the rich factor; the y-axis shows the pathway names)

A total of 148 and 149 KEGG pathways were obtained at 10 and 15 DPI, respectively. The top 20 pathways of KEGG pathway analysis of differentially expressed miRNA target genes are shown in Fig. 6 (Additional file 4). At 10 DPI, only 4 pathways were significantly enriched (*P* < 0.05). Of these, the Hedgehog signaling pathway was the most significantly enriched, which played an important role in the repair of injury and cell proliferation. At 15 DPI, only 3 pathways were significantly enriched (*P* < 0.05). Among them, Endocytosis and Phagosome were associated with defense responses against pathogenic microorganism.

### miRNA-gene network analysis

**Fig. 7:**
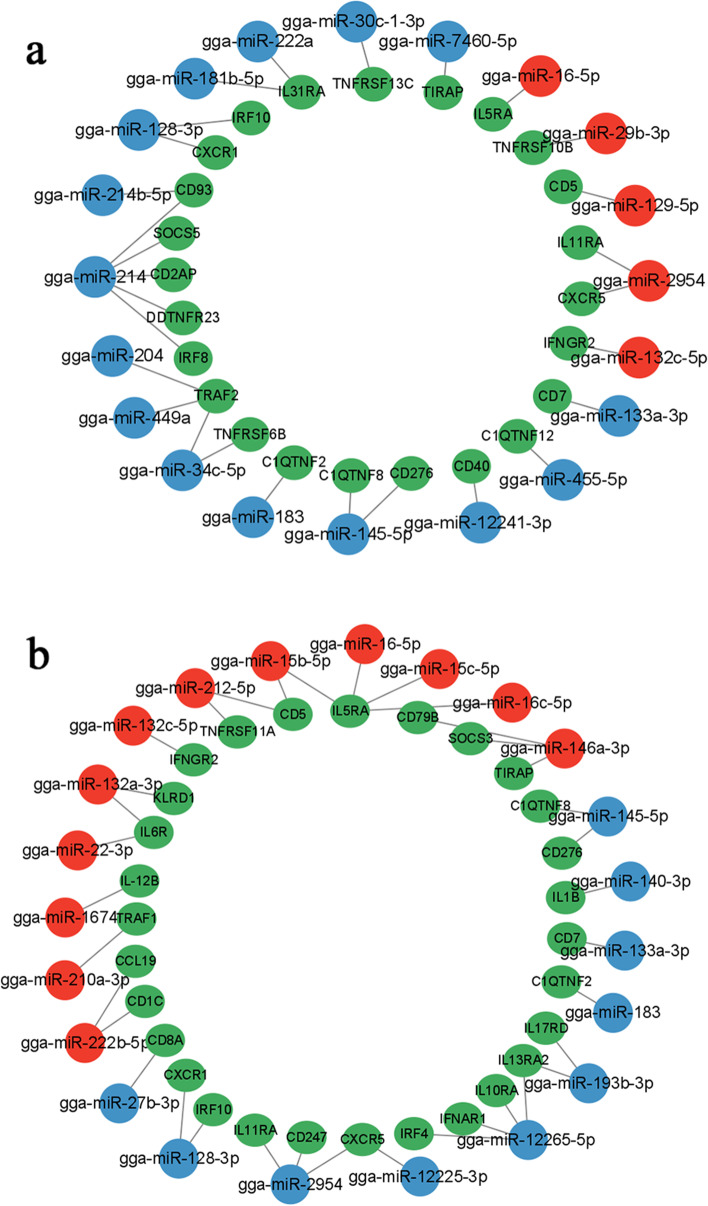
The network analysis of the interaction between the DEMs and their potential target genes. Red indicated up-regulated expressed miRNAs, blue indicated down-expressed miRNAs, and green indicated inflammatory and immunity-related target genes. **a, b** The interaction between the target genes and the DEMs in chicken cecum at 10 and 15 DPI

At 10 DPI (Fig. 7a), 24 different genes were possibly regulated by 20 DEMs. Among these genes, 6 target genes were regulated by up-regulated miRNA while 18 target genes were regulated by down-regulated miRNA. Among these DEMs, gga-miR-214 regulated the greatest number of target genes, with 5 targets. The target gene regulated by the greatest number of DEMs was TRAF2, with 3 miRNAs. At 15 DPI (Fig. 7b), 29 different genes were possibly regulated by 22 DEMs. Among these genes, 13 target genes were regulated by up-regulated miRNA while 16 target genes were regulated by down-regulated miRNA. Of these DEMs, gga-miR-12,265-5p regulated the greatest number of target genes, with 4 targets and followed by gga-miR-2954, regulated 3 targets. The target gene regulated by the greatest number of DEMs was IL5RA, with 4 miRNAs.

### Quantitative real-time qPCR validation

**Fig. 8:**
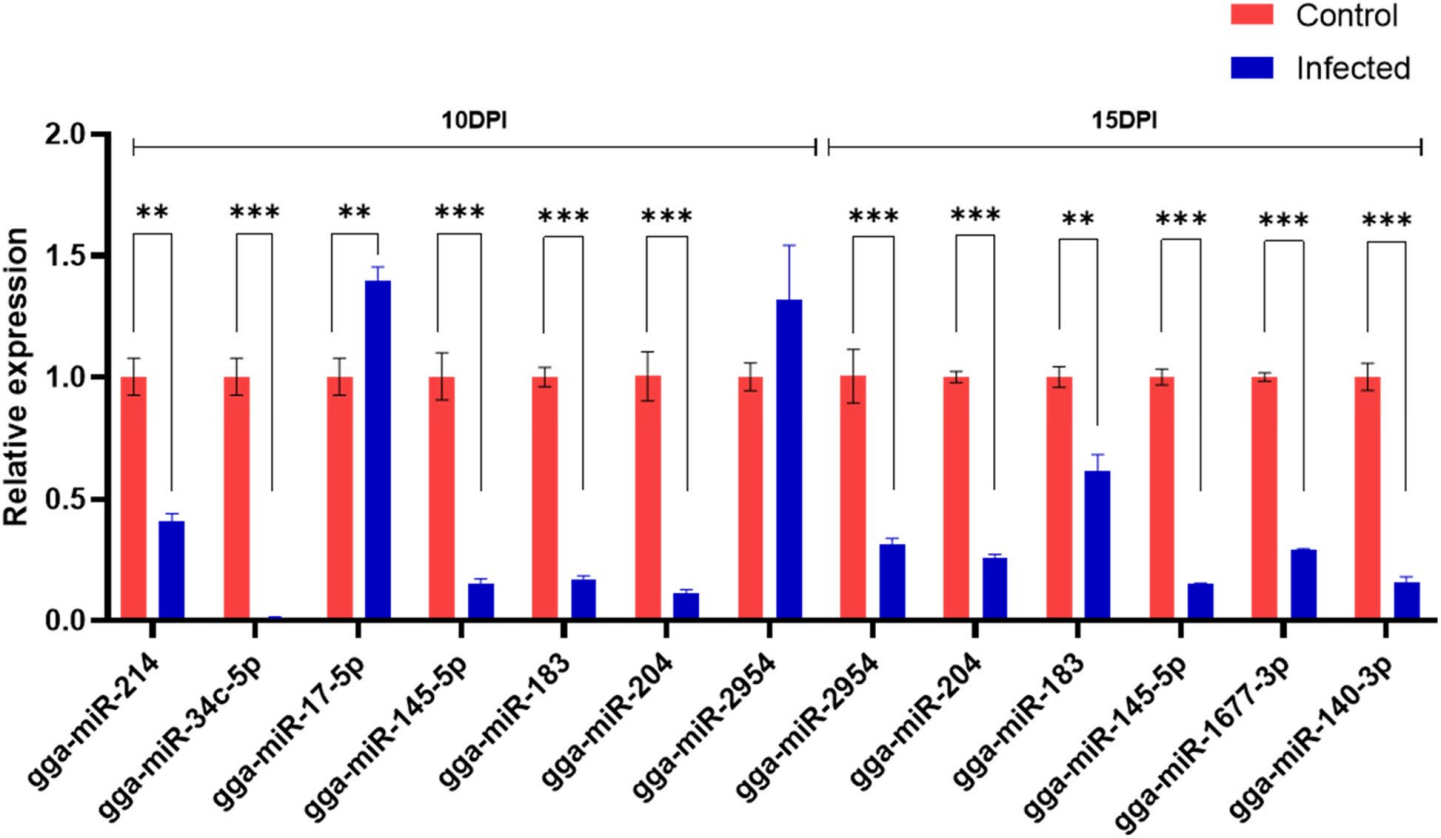
The expression level of differentially expressed miRNAs validated by RT-qPCR. MicroRNA expression was quantified relative to the expression level of U6 using the comparative cycle threshold (△CT) method. (* *P*<0.05, ** *P*<0.01, *** *P*<0.005)

The expression patterns of 9 miRNAs measured with RT-qPCR consistent with the high-throughput sequencing results (Fig. 8). The results verified the accuracy and reliability of the high-throughput sequencing results.

## Discussion

In this experiment, 94 and 127 miRNAs were identified as DEMs in chicken cecum samples at 10 and 15 DPI, respectively. It is obvious that more DEMs were identified in cecum samples at 15 DPI, and simultaneously, more severe pathological damage was also observed at this time point in this study. These not only represent the differences in the induced responses of the host between the two time points, but also imply that *H. meleagridis* infection may induce more biological processes involved in the host pathological formation at 15 DPI compared to 10 DPI. Among all of the identified DEMs, 60 DEMs were shared at 10 and 15 DPI. Interestingly, only one (gga-miR-2954) miRNA was up-regulated at 10 DPI and down-regulated at 15 DPI. Recent studies have revealed that miR-2954 plays an extensive regulatory role in normal development and disease [32–35]. And the other 59 DEMs showed the similar expression patterns at the two time points, indicating that these miRNAs were involved in the regulation of persistent infection of *H. meleagridis*. In addition, the numerous DEM homologs, including miR-29b-3p, miR-449a, let-7b, miR-146a-5p, miR-204, miR-128-3p, and miR-31-5p, miR-133a-3p, obtained from the chicken cecum in this study, has been shown to be associated with the intestinal mucosal integrity [36], intestinal inflammatory response [37–42], suggesting that these DEMs or DEM homologs may play the important roles in the cecum response against *H. meleagridis*. For instance, miR-133a-3p showed a downregulated expression in the cecum tissue of chickens following *H. meleagridis* infection at both time points, which was consistent with that in intestine of chicken coccidiosis [43], but showed an opposite expression pattern in cecum of chicken Salmonellosis [44]*.* A previous study found that miR-133a-3p inhibited the proliferation and promoted the apoptosis of intestinal epithelial cells by limiting the expression of TAGLN2 [36]. This indicates that miR-133a-3p might have a central role in chickens resistance to pathogenic, and could be responsible for the persistent inflammatory response and intestinal mucosal integrity in host cecum throughout the infection process.

At present, the research on host immune and inflammatory response caused by *H. meleagridis* infection was mainly focused on T cells, especially the immune and inflammatory response mediated by Th1 cells [45–47]. In this study, 27 GO terms related to T cell differentiation and function were screened to analyze the functions of the genes enriched into these terms (Additional file 3). The main findings of present work are summarized in Table 4. A large number of candidate target genes were involved in the promotion of Th1 and Th17 responses at both time points. In addition, we found that some candidate target genes inhibited Th2 responses at 10 DPI, while at 15 DPI, others candidate target genes promoted Th2 response. For example, RARA, having been shown to be involved in the differentiation of Th1 or Th2 cells [52, 58], was found to be potentially targeted by gga-miR-2954 that showed an up-regulated expression at 10 DPI and a down-regulated expression at 15 DPI. Moreover, PIPK2 [53], MALT1 [54], SOCS5 [50], STAT3 [51], IL12B [56], and IRF4 [57] has been shown to be associated with Th response. Among these, PIPK2 and MALT1 has been shown to be associated with promotion of Th1 and Th17 differentiation, respectively [53, 54]. STAT3 was capable of inhibiting Th2-mediated immune responses [51]. IRF4 has been shown to be related to promoting the differentiation of Th2 cell [57]. In this study, the potentially interactive combinations gga-miR-7460-3p/PIPK2 and gga-miR-145-5p/MALT1 at both time points, gga-miR-214/STAT3 at 10 DPI, and gga-miR-12,265-5p/IRF4 at 15 DPI may play the key roles in regulation of the Th cells responses in chicken cecum tissue infected with *H. meleagridis.* This showed that the host initiated different signaling pathways by the miRNAs and their targets to regulate Th cell differentiation to cope with the development of infection.

**Table 4:** Target genes and miRNA affection on Th cells at 10 and 15 DPI in GO terms

Infection time	miRNA	miRNA expression pattern	Target gene	Target gene affection on Th cells
10DPI	gga-miR-145-5p	down	CD276 [48] (B7-H3)	Inhibit Th2 Promote Th1/Th17
	gga-miR-214	down	LEF1 [49]	Inhibit Th2
	gga-miR-214	down	STAT3 [50]	Promote Th1/Th17
	gga-miR-214	down	SOCS5 [51]	Promote Th1
	gga-miR-2954	up	RARA [52]	Promote Th1
	gga-miR-7460-3p	down	RIPK2 [53]	Promote Th1/Th17
	gga-miR-145-5p	down	MALT1 [54]	Promote Th17
15DPI	gga-miR-7460-3p	down	RIPK2 [53]	Promote Th1/Th17
	gga-miR-145-5p	down	MALT1 [54]	Promote Th17
	gga-miR-148b-5p	down	STAT3 [50]	Promote Th1/Th17
	gga-miR-12,265-5p	down	RHOA [55]	Promote Th1/Th17
	gga-miR-1674	up	IL-12B [56]	Promote Th1
	gga-miR-12,265-5p	down	IRF4 [57]	Inhibit Th2
	gga-miR-2954	down	RARA [58]	Inhibit Th2

A total of 24 and 29 cytokines related to inflammation and immunity were selected at 10 and 15 DPI, respectively. The relationship between these target genes and Th cell and the results of these analyses are shown in Table 5. For example, IL11RA, having been shown to be involved in the differentiation of Th2 cells [59], was found to be potentially targeted by gga-miR-2954 that showed an up-regulated expression at 10 DPI and a down-regulated expression at 15 DPI. IL5RA [63], IFNGR2 [60], CD5 [61], CD2AP [68], CD276 [48], CCL19 [69], and TRAF1 [80] are involved in the regulation of Th cell responses. Among them, IL5RA has been shown to be associated with promote Th1 cell differentiation [63]. CD5 has been found to be involved in inhibiting Th2 cell differentiation [61]. TRAF1 has been shown to be associated with promote Th2 cell differentiation [80]. In this study, the potentially interactive combinations of gga-miR-16-5p/IL-5RA at both time points, gga-miR-129-5p/CD5 at 10 DPI, gga-miR-222b-5p/TRAF1 at 15 DPI may supports and extends the findings of GO enrichment analysis. These results may explain why more severe cecum lesions and inflammatory responses were observed from 10 to 15 days after infection, and why cecum lesions and inflammatory responses gradually abated from 15 days later.

**Table 5:** Cytokines and miRNA affection on Th cells at 10 and 15 DPI

Infection time	miRNA	miRNA expression pattern	Cytokine	Cytokine affection on Th cells
10DPI	gga-miR-2954	up	IL11RA [59]	Inhibit Th2
	gga-miR-16-5p	up	IL5RA [60]	Inhibit Th2
	gga-miR-129-5p	up	CD5 [61]	Inhibit Th2
	gga-miR-145-5p	down	CD276 [48] (B7-H3)	Inhibit Th2 Promote Th1/Th17
	gga-miR-7460-5p	down	TIRAP [62]	promote Th1
	gga-miR-132c-5p	up	IFNGR2 [63]	promote Th1
	gga-miR-214	down	SOCS5 [51]	promote Th1
	gga-miR-214	down	IRF8 [64–66]	promote Th1 Inhibit Th17/TFH
	gga-miR-214	down	CXCR5 [67]	Inhibit TFH
	gga-miR-2954	up	CD2AP [68]	Inhibit TFH
15DPI	gga-miR-2954, gga-miR-12,225-3p	down	IL11RA [59]	promote Th2
	gga-miR-222b-5p	up	TRAF1 [69]	promote Th2
	gga-miR-12,265-5p, gga-miR-193b-3p	down	IL13RA2 [70, 71]	Inhibit Th2 Promote Th1/Th17
	gga-miR-16-5p, gga-miR-15b-5p, gga-miR-15c-5p, gga-miR-16c-5p	up	IL5RA [72]	Inhibit Th2
	gga-miR-212-5p, gga-miR-15b-5p	up	CD5 [61]	Inhibit Th2
	gga-miR-146a-3p	up	SOCS3 [73]	Inhibit Th2
	gga-miR-132a-3p, gga-miR-22-3p	up	IL6R [74]	promote Th2 Inhibit Th1
	gga-miR-27b-3p	down	CD8A [75]	promote Th1
	gga-miR-132c-5p	up	IFNGR2 [63]	promote Th1
	gga-miR-1674	up	IL12B [56]	promote Th1
	gga-miR-193b-3p	down	IL17RD [76, 77]	promote Th17
	gga-miR-7460-5p	down	IL1B [78]	Promote Th1/Th17
	gga-miR-12,265-5p	down	IL10RA [79]	Inhibit Th1/Th17
	gga-miR-222b-5p	up	CCL19 [80]	Inhibit Th1
	gga-miR-146a-3p	up	TIRAP [62]	Inhibit Th1
	gga-miR-2954, gga-miR-12,225-3p	down	CXCR5 [60]	Promote TFH

In the KEGG pathway enrichment analysis of this study, in different periods after *H. meleagridis* infection, the host responds to the development of the disease by regulating different signal pathways (Additional file 4). The Hedgehog signaling pathway (Additional files 5, 7) at 10 DPI has been proved to play an important role in the development and function of the intestinal mucosa, gastrointestinal inflammation, and immune regulation [81, 82]. Activating the Hedgehog signal pathway inhibits the development of colitis by up-regulating the expression of anti-inflammatory cytokine IL-10 [83] while inhibiting the Hedgehog signal pathway lead to inflammatory bowel disease [84]. Gli1 and Gli2 has been shown to involved in activate the Hedgehog signal pathway to inhibit intestinal inflammation and balance inflammatory cytokines [85, 86]. In DEMs involved in the Hedgehog signaling pathway, gga-miR-6606-5p/Gli1 and gga-miR-7460-3p/Gli2 may have an important role in activating Hedgehog signaling pathway. The Phagosome (Additional files 6, 8) at 15 DPI has been shown to play an important role in the removal of pathogenic microorganisms [87, 88]. ITGB2 and SCARB1 capable of regulating the phagocytosis-promoting receptors on the surface of phagocytes [89]. In addition, SCARB1 has been shown to increase the number of anti-inflammatory macrophages and the expression of anti-inflammatory genes and is beneficial to tissue repair and regeneration [90]. RILP, M6PR, and CTSS has been demonstrated to participate in the fusion of phagosomes and late endosomes, the transport of cathepsin precursors, and the formation of cathepsin in lysosomes, respectively [91–93]. In DEMs involved in the Phagosome pathway, gga-miR-146c-5p/ ITGB2, gga-miR-140-3p/ SCARB1, gga-miR-145-5p/ RILP, gga-miR-148b-5p/ M6PR, and gga-miR-146a-3p/CTSS potentially involved with improved phagocytosis efficiency of Phagosome. This shows that the responses of host to *H. meleagridis* infection were different at 10 and 15 DPI, it mainly through the regulation of inflammatory responses at 10 DPI, while at 15 DPI, it could be mainly to remove *H. meleagridis*.

## Conclusions

At 10 and 15 DPI, a total of 161 DEMs were found, many of which are known to regulate host immune and inflammatory responses. Compared with 10 DPI, more DEMs were found at 15 DPI. KEGG enrichment analysis showed that the responses of the body to *H. meleagridis* infection were different at 10 and 15 DPI. GO enrichment analysis and miRNA-gene network analysis revealed that the immune response of the host caused by *H. meleagridis* infection is not limited to Th1.

## Supplementary Information

**Additional file 1: Table S1.** Differentially expressed miRNAs between CE and CC samples

**Additional file 2: Table S2.** Differentially expressed miRNAs between CEH and CCH samples

**Additional file 3: Table S3.** T cell related GO terms of target genes of DEMs obtained from the samples at 10 and 15 DPI

**Additional file 4: Table S4.** KEGG pathways of target genes of DEMs obtained from the samples at 10 and 15 DPI

**Additional file 5: Table S5.** Differentially expressed miRNAs induced by *H. meleagridis* at 10 DPI regulating the gene expression in Hedgehog signaling pathway

**Additional file 6: Table S6.** Differentially expressed miRNAs induced by *H. meleagridis* at 15 DPI regulating the gene expression in Endocytosis and Phagosome pathway

**Additional file 7.**

**Additional file 8.**

## Data Availability

The datasets supporting the findings of this article are included within the article. All sequence data was submitted to the NCBI Gene Expression Omnibus (GEO) public database (http://www.ncbi.nlm.nih.gov/geo/) with the GEO accession number GSE193859.

## Abbreviations

DEMs: Differentially expressed miRNAs
TPM: The number of transcripts per million clean tags
DPI: day Post-infection
GEO: Gene Expression Omnibus
GO: Gene ontology
IL: Interleukin
ILR: Interleukin receptor
KEGG: Kyoto Encyclopedia of Genes and Genomes
RT-qPCR: Quantitative reverse transcription real-time PCR
sRNA: Small RNA
TNF: Tumor necrosis factors
TRAF: TNF receptor-associated factor
YY1: Yin Yang1
PI3K: phosphoinositide 3-kinases
CLIC4: chloride intracellular channel protein 4
NLRP3: leucine-rich repeat-containing protein 3
MMP8: matrix Metallo proteinase-8X
NF-_Κ_B: nuclear factor kappa-light-chain-enhancer of activated B cells
Th: T-helper
TFH: follicular helper T
LEF1: Lymphoid enhancer binding factor-1
STAT: signal transducer and activator of transcription
SOCS: suppressor of cytokine signaling
RARA: Retinoic acid receptor alpha
RIPK2: threonine-protein kinase-2
MALT1: Mucosa-associated lymphoid tissue lymphoma translocation protein 1
RHOA: The small GTPase
IFN: Interferons
IRF: interferon regulatory factor
IFNGR2: interferon-gamma receptor 2
TIRAP: Toll/interleukin-1 receptor adapter protein
CXCR: C-X-C chemokine receptor type
CD2AP: CD2 associated protein
CD8A: cluster of differentiation 8a
CCL19: CC-chemokine ligand 19
Gli: glioma associated oncogene
ITGB2: integrin subunit beta 2
SCARB1: scavenger receptor class B member I
RILP: Rab-interacting lysosomal protein
M6PR: Mannose-6-phosphate reductase
CTSS: Cathepsin S

## References

[1] Tyzzer EE (1920). The Flagellate Character and Reclassification of the Parasite Producing "Blackhead" in Turkeys: Histomonas (Gen. nov.) meleagridis (Smith). J Parasitol.

[2] McDougald LR (2005). Blackhead disease (histomoniasis) in poultry: a critical review. Avian Dis.

[3] Graybill HW, Smith T (1920). Production Of Fatal Blackhead In Turkeys By Feeding Embryonated Eggs Of Heterakis Papillosa. J Exp Med.

[4] Beckmann JF, Dormitorio T, Oladipupo SO, Bethonico Terra MT, Lawrence K, Macklin KS, Hauck R (2021). Heterakis gallinarum and Histomonas meleagridis DNA persists in chicken houses years after depopulation. Vet Parasitol.

[5] Liu D, Kong L, Tao J, Xu J (2018). An Outbreak of Histomoniasis in Backyard Sanhuang Chickens. Korean J Parasitol.

[6] Windisch M, Hess M (2010). Experimental infection of chickens with Histomonas meleagridis confirms the presence of antibodies in different parts of the intestine. Parasite Immunol.

[7] Liebhart D, Ganas P, Sulejmanovic T, Hess M (2017). Histomonosis in poultry: previous and current strategies for prevention and therapy. Avian Pathol.

[8] Xu J, Qu C, Guo P, Zhuo Z, Liu D, Tao J (2018). Epidemic Characteristics of Clinical Histomoniasis in Chicken Flocks in Eastern China. Avian Dis.

[9] Hess M, Liebhart D, Bilic I, Ganas P (2015). Histomonas meleagridis—New insights into an old pathogen. Vet Parasitol.

[10] Molnár A, Schwach F, Studholme DJ, Thuenemann EC, Baulcombe DC (2007). miRNAs control gene expression in the single-cell alga Chlamydomonas reinhardtii. NATURE..

[11] van Rooij E (2011). The Art of MicroRNA Research. Circ Res.

[12] Liu J, Li F, Hu X, Cao D, Liu W, Han H, Zhou Y, Lei Q (2021). Deciphering the miRNA transcriptome of breast muscle from the embryonic to post-hatching periods in chickens. BMC Genomics.

[13] Wang W, Yang Q, Huang X, Luo R, Xie K, Gao X, Yan Z, Wang P, Zhang J, Yang J (2021). Effects of miR-204 on apoptosis and inflammatory response of Clostridium perfringens beta2 toxin induced IPEC-J2 cells via targeting BCL2L2. Microb Pathogenesis.

[14] Wang Q, Liu Y, Wu Y, Wen J, Man C (2021). Immune function of miR-214 and its application prospects as molecular marker. PEERJ..

[15] Lian L, Zhang D, Wang Q, Yang N, Qu L (2015). The inhibitory effects of gga-miR-199-3p, gga-miR-140-3p, and gga-miR-221-5p in Marek's disease tumorigenesis. Poult Sci.

[16] Hong YH, Dinh H, Lillehoj HS, Song K, Oh J (2014). Differential regulation of microRNA transcriptome in chicken lines resistant and susceptible to necrotic enteritis disease. Poult Sci.

[17] Hou Z, Liu D, Su S, Wang L, Zhao Z, Ma Y, Li Q, Jia C, Xu J, Zhou Y (2019). Comparison of splenocyte microRNA expression profiles of pigs during acute and chronic toxoplasmosis. BMC Genomics.

[18] Clarke LL, Beckstead RB, Hayes JR, Rissi DR (2017). Pathologic and molecular characterization of histomoniasis in peafowl (Pavo cristatus). J Vet Diagn Invest.

[19] Beckmann JF, Dormitorio T, Oladipupo SO, Bethonico Terra MT, Lawrence K, Macklin KS, Hauck R (2021). Heterakis gallinarum and Histomonas meleagridis DNA persists in chicken houses years after depopulation. Vet Parasitol.

[20] Pham AD, Mast J, Magez S, Goddeeris BM, Carpentier SC (2016). The Enrichment of Histomonas meleagridis and Its Pathogen-Specific Protein Analysis: A First Step to Shed Light on Its Virulence. Avian Dis.

[21] Chen C, Chen QG, Wang S, Rong J, Liu DD, Hou ZF, Tao JP, Xu JJ (2021). Prokaryotic expression and localization analysis of α-actinin 1 protein of *Histomonas meleagridis*. Chin. J. Prev. Vet. Med..

[22] Langmead B, Trapnell C, Pop M, Salzberg SL (2009). Ultrafast and memory-efficient alignment of short DNA sequences to the human genome. Genome Biol.

[23] Friedländer MR, Mackowiak SD, Li N, Chen W, Rajewsky N (2012). miRDeep2 accurately identifies known and hundreds of novel microRNA genes in seven animal clades. Nucleic Acids Res.

[24] Wen M, Shen Y, Shi S, Tang T (2012). miREvo: an integrative microRNA evolutionary analysis platform for next-generation sequencing experiments. BMC BIOINFORMATICS.

[25] Zhou L, Chen J, Li Z, Li X, Hu X, Huang Y, Zhao X, Liang C, Wang Y (2010). Integrated profiling of microRNAs and mRNAs: microRNAs located on Xq27.3 associate with clear cell renal cell carcinoma. PLoS One.

[26] Ferreira JA, Zwinderman AH (2006). On the Benjamini–Hochberg method. Ann Stat.

[27] Enright AJ, John B, Gaul U, Tuschl T, Sander C, Marks DS (2003). MicroRNA targets in Drosophila. Genome Biol.

[28] Kruger J, Rehmsmeier M (2006). RNAhybrid: microRNA target prediction easy, fast and flexible. Nucleic Acids Res.

[29] Young MD, Wakefield MJ, Smyth GK, Oshlack A (2010). Gene ontology analysis for RNA-seq: accounting for selection bias. Genome Biol.

[30] Kanehisa M, Araki M, Goto S, Hattori M, Hirakawa M, Itoh M, Katayama T, Kawashima S, Okuda S, Tokimatsu T (2007). KEGG for linking genomes to life and the environment. Nucleic Acids Res.

[31] Mao X, Cai T, Olyarchuk JG, Wei L (2005). Automated genome annotation and pathway identification using the KEGG Orthology (KO) as a controlled vocabulary. BIOINFORMATICS..

[32] Dong X, Cheng Y, Qiao L, Wang X, Zeng C, Feng Y (2021). Male-Biased gga-miR-2954 Regulates Myoblast Proliferation and Differentiation of Chicken Embryos by Targeting YY1. GENES-BASEL..

[33] Li J, Li C, Li Q, Li W, Li H, Li G, Kang X, Liu X, Tian Y (2020). Identification of the Key microRNAs and miRNA-mRNA Interaction Networks during the Ovarian Development of Hens. ANIMALS..

[34] Tian H, Ding M, Guo Y, Su A, Zhai M, Tian Y, Li K, Sun G, Jiang R, Han R (2021). Use of transcriptomic analysis to identify microRNAs related to the effect of stress on thymus immune function in a chicken stress model. Res Vet Sci.

[35] Liu Q, Cai J, Gao Y, Yang J, Gong Y, Zhang Z (2018). miR-2954 Inhibits PI3K Signaling and Induces Autophagy and Apoptosis in Myocardium Selenium Deficiency. Cell Physiol Biochem.

[36] Tian X, Li L, Fu G, Wang J, He Q, Zhang C, Qin B, Wang J (2021). miR-133a-3p regulates the proliferation and apoptosis of intestinal epithelial cells by modulating the expression of TAGLN2. Exp Ther Med.

[37] Dai Y, Mao Z, Han X, Xu Y, Xu L, Yin L, Qi Y, Peng J (2019). MicroRNA-29b-3p reduces intestinal ischaemia/reperfusion injury via targeting of TNF receptor-associated factor 3. Br J Pharmacol.

[38] Zhao D, Wu N, Wang L, Pang X, Liu X, Zhang X (2020). Role of microRNA-449a in the progress of inflammatory bowel disease in children. Biotechnol. Biotechnol. Equip.

[39] Liu Z, Tian Y, Jiang Y, Chen S, Liu T, Moyer MP, Qin H, Zhou X (2018). Protective Effects of Let-7b on the Expression of Occludin by Targeting P38 MAPK in Preventing Intestinal Barrier Dysfunction. Cell Physiol Biochem.

[40] Chen J, Chen T, Zhou J, Zhao X, Sheng Q, Lv Z (2021). MiR-146a-5p Mimic Inhibits NLRP3 Inflammasome Downstream Inflammatory Factors and CLIC4 in Neonatal Necrotizing Enterocolitis. Front Cell Dev Biol.

[41] Chen G, Han Y, Feng Y, Wang A, Li X, Deng S, Zhang L, Xiao J, Li Y, Li N (2019). Extract of Ilex rotunda Thunb alleviates experimental colitis-associated cancer via suppressing inflammation-induced miR-31-5p/YAP overexpression. PHYTOMEDICINE..

[42] Huo LL, Sun ZR (2021). MiR-128-3p alleviates TNBS-induced colitis through inactivating TRAF6/NF-κB signaling pathway in rats. Kaohsiung J Med Sci.

[43] Tim G, Tommy VL, Panagiotis S, Lily Q, Aouatif B, Dominiek M, Ilias K, Paul B, Neil F (2020). Diagnosis of sub-clinical coccidiosis in fast growing broiler chickens by MicroRNA profiling. GENOMICS..

[44] Wu G, Qi Y, Liu X, Yang N, Xu G, Liu L, Li X (2017). Cecal MicroRNAome response to Salmonella enterica serovar Enteritidis infection in White Leghorn Layer. BMC Genomics.

[45] Kidane FA, Mitra T, Wernsdorf P, Hess M, Liebhart D (2018). Allocation of Interferon Gamma mRNA Positive Cells in Caecum Hallmarks a Protective Trait Against Histomonosis. Front Immunol.

[46] Lagler J, Mitra T, Schmidt S, Pierron A, Vatzia E, Stadler M (2019). Cytokine production and phenotype of Histomonas meleagridis-specific T cells in the chicken. Vet Res.

[47] Lagler J, Schmidt S, Mitra T, Stadler M, Wernsdorf P, Grafl B, Hess THC, Gerner W, Liebhart D (2021). Comparative investigation of IFN-gamma-producing T cells in chickens and turkeys following vaccination and infection with the extracellular parasite Histomonas meleagridis. Dev Comp Immunol.

[48] Luo L, Zhu G, Xu H, Yao S, Zhou G, Zhu Y, Tamada K, Huang L, Flies AD, Broadwater M (2015). B7-H3 Promotes Pathogenesis of Autoimmune Disease and Inflammation by Regulating the Activity of Different T Cell Subsets. PLoS One.

[49] Uematsu Y, Akai S, Tochitani T, Oda S, Yamada T, Yokoi T (2016). MicroRNA-mediated Th2 bias in methimazole-induced acute liver injury in mice. Toxicol Appl Pharmacol.

[50] Shi Y, Dai S, Qiu C, Wang T, Zhou Y, Xue C, Yao J, Xu Y (2020). MicroRNA-219a-5p suppresses intestinal inflammation through inhibiting Th1/Th17-mediated immune responses in inflammatory bowel disease. Mucosal Immunol.

[51] Seki Y, Hayashi K, Matsumoto A, Seki N, Tsukada J, Ransom J, Naka T, Kishimoto T, Yoshimura A, Kubo M (2002). Expression of the Suppressor of Cytokine Signaling-5 (SOCS5) Negatively Regulates IL-4-Dependent STAT6 Activation and Th2 Differentiation. Proc Natl Acad Sci U S A.

[52] Bene K, Varga Z, Petrov VO, Boyko N, Rajnavolgyi E (2017). Gut Microbiota Species Can Provoke both Inflammatory and Tolerogenic Immune Responses in Human Dendritic Cells Mediated by Retinoic Acid Receptor Alpha Ligation. Front Immunol.

[53] Cirauqui C, Benito-Villalvilla C, Sanchez-Ramon S, Sirvent S, Diez-Rivero C (2018). Human dendritic cells activated with MV130 induce Th1, Th17 and IL-10 responses via RIPK2 and MyD88 signalling pathways. Eur J Immunol.

[54] Chen X, Zhang X, Lan L, Xu G, Li Y, Huang S (2021). MALT1 positively correlates with Th1 cells, Th17 cells, and their secreted cytokines and also relates to disease risk, severity, and prognosis of acute ischemic stroke. J Clin Lab Anal.

[55] Pan W, Nagpal K, Suarez-Fueyo A, Ferretti A, Yoshida N, Tsokos M, Tsokos G (2021). The Regulatory Subunit PPP2R2A of PP2A Enhances Th1 and Th17 Differentiation through Activation of the GEF-H1/RhoA/ROCK Signaling Pathway. J Immunol.

[56] Shiraki M, Aihara H, Kinouchi Y, Takahashi S, Oki M, Noguchi M, Takahashi K, Miyazaki J, Shimosegawa T (2004). IL-12 p40 prevents the development of chronic enterocolitis in IL-10-deficient mice. Lab Invest.

[57] Williams JW, Tjota MY, Clay BS, Vander Lugt B, Bandukwala HS, Hrusch CL (2013). Transcription factor IRF4 drives dendritic cells to promote Th2 differentiation. Nat Commun.

[58] Yuan X, Tang H, Wu R, Li X, Jiang H, Liu Z, Zhang Z (2021). Short-Chain Fatty Acids Calibrate RARalpha Activity Regulating Food Sensitization. Front Immunol.

[59] Curti A, Ratta M, Corinti S, Girolomoni G, Ricci F, Tazzari P, Siena M (2001). Interleukin-11 induces Th2 polarization of human CD4(+) T cells. BLOOD..

[60] Narayanan S, Lee J, Bhagwate A, Kuwelker S, Yan H, Ordog T, Bharucha A (2020). Epigenetic Alterations Are Associated With Gastric Emptying Disturbances in Diabetes Mellitus. CLIN TRANSL GASTROEN.

[61] Sestero CM, McGuire DJ, De Sarno P, Brantley EC, Soldevila G, Axtell RC, Raman C (2012). CD5-dependent CK2 activation pathway regulates threshold for T cell anergy. J Immunol.

[62] Imanishi T, Unno M, Kobayashi W, Yoneda N, Akira S, Saito T (2020). mTORC1 Signaling Controls TLR2-Mediated T-Cell Activation by Inducing TIRAP Expression. Cell Rep.

[63] Tau GZ, von der Weid T, Lu B, Cowan S, Kvatyuk M, Pernis A, Cattoretti G (2000). Interferon gamma signaling alters the function of T helper type 1 cells. J Exp Med.

[64] Luda KM, Joeris T, Persson EK, Rivollier A, Demiri M, Sitnik KM, Pool L, Holm JB, Melo-Gonzalez F, Richter L (2016). IRF8 Transcription-Factor-Dependent Classical Dendritic Cells Are Essential for Intestinal T Cell Homeostasis. IMMUNITY..

[65] Zhang R, Qi C, Hu Y, Shan Y, Hsieh Y, Xu F, Lu G, Dai J, Gupta M, Cui M (2019). T follicular helper cells restricted by IRF8 contribute to T cell-mediated inflammation. J Autoimmun.

[66] Ouyang X, Zhang R, Yang J, Li Q, Qin L, Zhu C, Liu J, Ning H, Shin MS, Gupta M (2011). Transcription factor IRF8 directs a silencing programme for TH17 cell differentiation. Nat Commun.

[67] Yu D, Rao S, Tsai LM, Lee SK, He Y, Sutcliffe EL, Srivastava M, Linterman M, Zheng L, Simpson N (2009). The Transcriptional Repressor Bcl-6 Directs T Follicular Helper Cell Lineage Commitment. Immunity (Cambridge, Mass).

[68] Raju S, Kometani K, Kurosaki T, Shaw AS, Egawa T (2018). The adaptor molecule CD2AP in CD4 T cells modulates differentiation of follicular helper T cells during chronic LCMV infection. PLoS Pathog.

[69] Bryce PJ, Oyoshi MK, Kawamoto S, Oettgen HC, Tsitsikov EN (2006). TRAF1 regulates Th2 differentiation, allergic inflammation and nuclear localization of the Th2 transcription factor, NIP45. Int Immunol.

[70] Yasunaga SI, Yuyama N, Arima K, Tanaka H, Toda S, Maeda M, Matsui K, Goda C, Yang Q, Sugita Y (2003). The negative-feedback regulation of the IL-13 signal by the IL-13 receptor α2 chain in bronchial epithelial cells. CYTOKINE..

[71] Wilson MS, Ramalingam TR, Rivollier A, Shenderov K, Mentink Kane MM, Madala SK, Cheever AW, Artis D, Kelsall BL, Wynn TA (2011). Colitis and Intestinal Inflammation in IL10−/− Mice Results From IL-13Rα2–Mediated Attenuation of IL-13 Activity. GASTROENTEROLOGY..

[72] Hosokawa H, Kato M, Tohyama H, Tamaki Y, Endo Y, Kimura MY, Tumes DJ, Motohashi S, Matsumoto M, Nakayama KI (2015). Methylation of Gata3 Protein at Arg-261 Regulates Transactivation of the Il5 Gene in T Helper 2 Cells. J Biol Chem.

[73] Kubo M, Inoue H (2006). Suppressor of cytokine signaling 3 (SOCS3) in Th2 cells evokes Th2 cytokines, IgE, and eosinophilia. Curr Allergy Asthma Rep.

[74] Guerrero AR, Uchida K, Nakajima H, Watanabe S, Nakamura M, Johnson WE, Baba H (2012). Blockade of interleukin-6 signaling inhibits the classic pathway and promotes an alternative pathway of macrophage activation after spinal cord injury in mice. J Neuroinflammation.

[75] Li Y, Liu X, Duan W, Tian H, Zhu G, He H, Yao S, Yi S, Song W, Tang H (2017). Batf3-dependent CD8α + Dendritic Cells Aggravates Atherosclerosis via Th1 Cell Induction and Enhanced CCL5 Expression in Plaque Macrophages. EBIOMEDICINE..

[76] Su Y, Huang J, Zhao X, Lu H, Wang W, Yang XO, Shi Y, Wang X, Lai Y, Dong C (2019). Interleukin-17 receptor D constitutes an alternative receptor for interleukin-17A important in psoriasis-like skin inflammation. Science immunology.

[77] Shapiro M, Nandi B, Gonzalez G, Prabhala RH, Mashimo H, Huang Q, Frank NY, Munshi NC, Gold JS (2017). Deficiency of the immunostimulatory cytokine IL-21 promotes intestinal neoplasia via dysregulation of the Th1/Th17 axis. ONCOIMMUNOLOGY..

[78] Uchiyama R, Yonehara S, Taniguchi SI, Ishido S, Ishii KJ, Tsutsui H (2017). Inflammasome and Fas-Mediated IL-1β Contributes to Th17/Th1 Cell Induction in Pathogenic Bacterial Infection In Vivo. J Immunol.

[79] Veenbergen S, van Leeuwen MA, Driessen GJ, Kersseboom R, de Ruiter LF, Raatgeep RHC, Lindenbergh-Kortleve DJ, Simons-Oosterhuis Y, Biermann K, Halley DJJ (2017). Development and Function of Immune Cells in an Adolescent Patient With a Deficiency in the Interleukin-10 Receptor. J Pediatr Gastroenterol Nutr.

[80] Marsland BJ, Bättig P, Bauer M, Ruedl C, Lässing U, Beerli RR, Dietmeier K (2005). CCL19 and CCL21 Induce a Potent Proinflammatory Differentiation Program in Licensed Dendritic Cells. Immunity (Cambridge, Mass).

[81] Lees C, Howie S, Sartor RB, Satsangi J (2005). The Hedgehog Signalling Pathway in the Gastrointestinal Tract: Implications for Development, Homeostasis, and Disease. GASTROENTEROLOGY..

[82] Xie Z, Zhang M, Zhou G, Lin L, Han J, Wang Y, Li L, He Y, Zeng Z, Chen M (2021). Emerging roles of the Hedgehog signalling pathway in inflammatory bowel disease. Cell Death Dis.

[83] Lee JJ, Rothenberg ME, Seeley ES, Zimdahl B, Kawano S, Lu W, Shin K, Sakata-Kato T, Chen JK, Diehn M (2016). Control of inflammation by stromal Hedgehog pathway activation restrains colitis. Proc Natl Acad Sci.

[84] Buongusto F, Bernardazzi C, Yoshimoto AN, Nanini HF, Coutinho RL, Carneiro AJV, Castelo-Branco MT, de Souza HS (2017). Disruption of the Hedgehog signaling pathway in inflammatory bowel disease fosters chronic intestinal inflammation. Clin Exp Med.

[85] Lees CW, Zacharias WJ, Tremelling M, Noble CL, Nimmo ER, Tenesa A, Cornelius J, Torkvist L, Kao J, Farrington S (2008). Analysis of germline GLI1 variation implicates hedgehog signalling in the regulation of intestinal inflammatory pathways. PLoS Med.

[86] Liu Z, Lai K, Xie Y, He X, Zhou X (2018). Gli2 Mediated Activation of Hedgehog Signaling Attenuates Acute Pancreatitis via Balancing Inflammatory Cytokines in Mice. Cell Physiol Biochem.

[87] Scianimanico S, Desrosiers M, Dermine JF, Meresse S, Descoteaux A, Desjardins M (1999). Impaired recruitment of the small GTPase rab7 correlates with the inhibition of phagosome maturation by Leishmania donovani promastigotes. Cell Microbiol.

[88] Marsman M, Jordens I, Kuijl C, Janssen L, Neefjes J (2004). Dynein-mediated vesicle transport controls intracellular Salmonella replication. Mol Biol Cell.

[89] Underhill DM, Ozinsky A (2002). Phagocytosis of Microbes: Complexity in Action. Annu Rev Immunol.

[90] Zhang J, Qu C, Li T, Cui W, Wang X, Du J (2019). Phagocytosis mediated by scavenger receptor class BI promotes macrophage transition during skeletal muscle regeneration. J Biol Chem.

[91] Harrison RE, Bucci C, Vieira OV, Schroer TA, Grinstein S (2003). Phagosomes Fuse with Late Endosomes and/or Lysosomes by Extension of Membrane Protrusions along Microtubules: Role of Rab7 and RILP. Mol Cell Biol.

[92] Zeng GZ, Tan NH, Jia RR, Pan XL (2005). Cathepsins: Structures, Functions and Inhibitors. Acta Botanica Yunnanica.

[93] Pires D, Bernard EM, Pombo JP, Carmo N, Fialho C, Gutierrez MG, Bettencourt P, Anes E (2017). Mycobacterium tuberculosis Modulates miR-106b-5p to Control Cathepsin S Expression Resulting in Higher Pathogen Survival and Poor T-Cell Activation. Front Immunol.

