# Supplementary figures and images for: MicroRNA expression profile of chicken cecum in different stages during Histomonas meleagridis infection
Source: BMC Vet Res. 2022 Jun 11;18:222. doi: 10.1186/s12917-022-03316-2 (PMC9188098; doi:10.1186/s12917-022-03316-2)

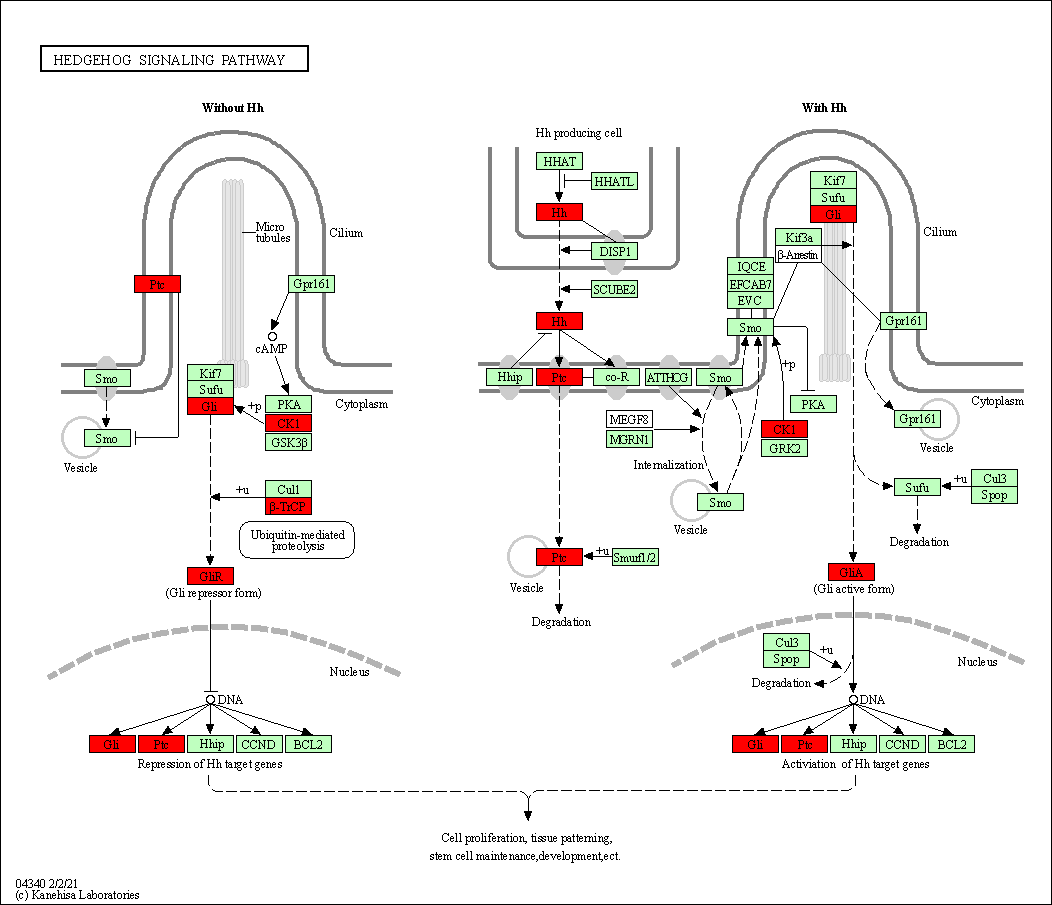

Supplement: Supplementary file 7 — Additional file 7. [file 12917_2022_3316_MOESM7_ESM.png]

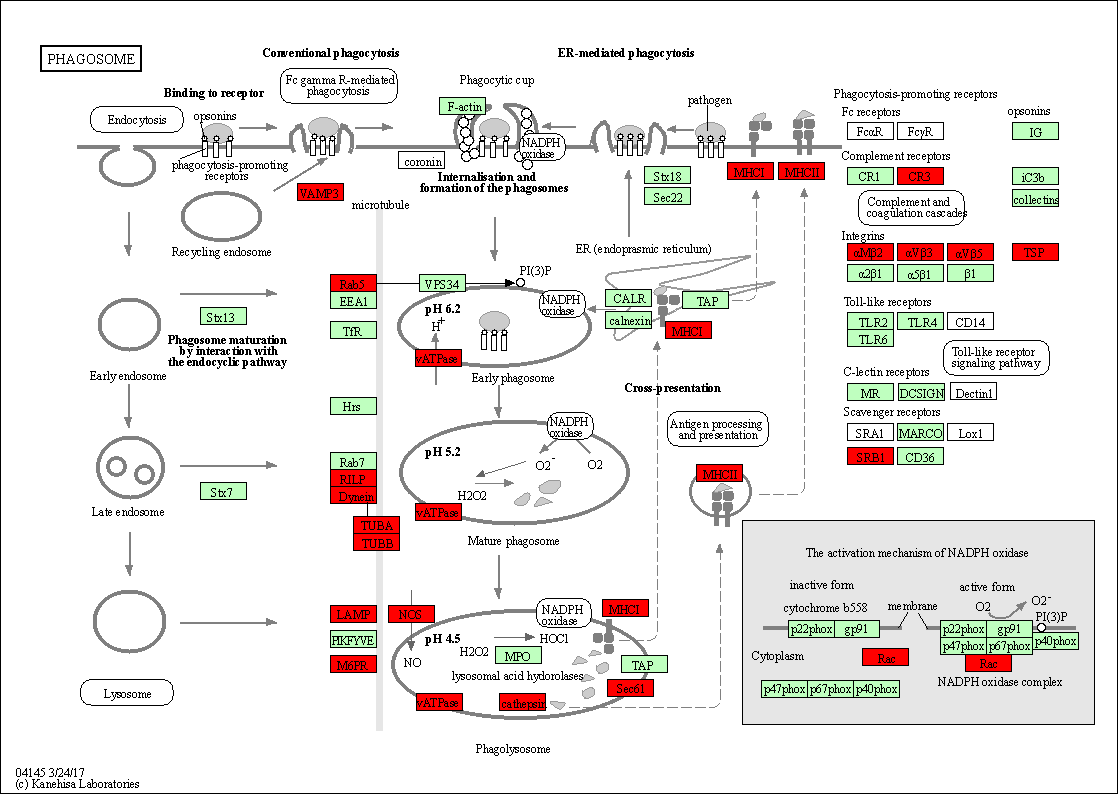

Supplement: Supplementary file 8 — Additional file 8. [file 12917_2022_3316_MOESM8_ESM.png]
